# The Effect of Intruder Density on Territoriality and Dominance in Male Swimming Crab (*Portunus trituberculatus*)

**DOI:** 10.3390/ani12030314

**Published:** 2022-01-27

**Authors:** Boshan Zhu, Hanzun Zhang, Yunliang Lu, Fang Wang, Dapeng Liu

**Affiliations:** 1 The Key Laboratory of Mariculture, Ministry of Education, Ocean University of China, 5 Yushan Road, Qingdao 266003, China; zhuboshan@stu.ouc.edu.cn (B.Z.); m18660282628_1@163.com (H.Z.); wangfang249@ouc.edu.cn (F.W.); 2 Function Laboratory for Marine Fisheries Science and Food Production Processes, Qingdao National Laboratory for Marine Science and Technology, 1 Wenhai Road, Qingdao 266003, China; 3 School of Marine Science and Engineering, Qingdao Agricultural University, Qingdao 266109, China; yun.2004@163.com

**Keywords:** intruder number, *Portunus trituberculatus*, territorial behavior, dominance hierarchy, population quantity

## Abstract

**Simple Summary:**

Territorial behavior is closely correlated with population fluctuations in territorial species, which is influenced by the density of conspecifics. Relevant research in aquacultural species, such as swimming crab (*Portunus trituberculatus*), is still lacking. In this study, we quantified the territorial behavior of the crabs according to a different number of intruders. This study provides a behavioral perspective for understanding and predicting the population dynamics of marine benthonic animals.

**Abstract:**

Territorial behavior of animals is affected by numerous factors, one being the number of intruders. The swimming crab (*Portunus trituberculatus*), an important commercial and ecological species on the continental shelf of Asia, usually needs to defend its territory from intrusion by other crabs, especially in habitats with high densities of conspecifics. To clarify the underlying patterns of how *P. trituberculatus* protects its territory, we assessed the territorial behavior of occupant crabs (territory holders) when presented with different numbers of intruders using an indoor observation system. We calculated the territory size of the occupants and quantified their behavioral responses to intruders. With an increased number of intruders, the territory size the occupants owned significantly decreased, and their behavior adjusted accordingly. Besides, the territorial behavior score, reflecting the territoriality of crab, decreased significantly. Furthermore, in a high density group that had seven intruders, the occupants showed a higher dominance hierarchy than the intruders, indicating the ascendancy of occupants in territorial competition with intruders. These results revealed that as the number of intruders increased, the territory size of *P. trituberculatus* shrunk because the fight for territory became more intense.

## 1. Introduction

Territory is an essential resource that many animals require to survive, grow, and reproduce [1]. Territorial behavior, including patrolling and exhibiting aggression towards intruders, is an important feature of territorial species [2,3,4]. Individuals with territories have a greater chance of survival because they can more effectively monopolize limited resources including food, shelter, and mates within their territory [5]. For example, crayfish (*Orconectes propinquus*) occupying a territory may acquire increased access to shelter and food resources [6]. Nevertheless, as the territory expands, there will be more intrusions from others, especially from the conspecifics due to the high resource use overlapping within a similar size or age class [7]. Frequent intrusions usually cause the occupants to incur higher costs, in terms of time, energy, injury risk, and even death, in expelling intruders and maintaining a dominant position [8,9,10]. Accordingly, the number of intruders is a vital regulator affecting the territory quality and the territory size that an animal can have [11,12].

The contender pressure hypothesis suggests that there is a negative correlation between the territory size and conspecific density [12]. The intrusion and expulsion on territory will be more intensify as the density of conspecifics increases [12]. Additionally, the net benefits of occupants rapidly decline as maintenance costs increase [13,14]. This hypothesis has been tested in some aquatic species such as the crayfish (*Orconenectes propinquus*) and Mediterranean trout (*Salmo trutta*) [6,15]. Within territories, dominance hierarchies may develop after individuals repeatedly interact with one another.

A dominance hierarchy is developed after individuals repeatedly interact [16]. During competition, the winner becomes the dominant individual and the loser becomes the subordinate individual [17]. These relationships are retained and lay a foundation for the development of the dominance hierarchy in the population, which closely reduces the frequency of fighting for resource and contributes to the explanation of population fluctuations [16,17,18]. Research into dominance hierarchies in crustaceans has generally focused on the influences of resource types including females and food [17,19], and rarely addresses the relationship with territory, although the dominance hierarchy between occupants and intruders may regulate the territorial behavior by affecting the prior-residence effect [20,21]. This effect demonstrates that the occupants usually have a strong internal motivation to expel intruders and are more likely to gain advantages in such contests [6]. However, as previously mentioned, the increasing number of intruders may reduce the benefits of protecting the territory of the occupants [22], forcing them to decide whether to continue to maintain or abandon their territory once the benefits fall below a certain threshold [16,23]. Despite the related discussions that are arising, the connections between dominance hierarchy and territory in aquatic crustaceans are still poorly understood.

The swimming crab (*Portunus trituberculatus*) is a territorial species in the shelf of West Pacific Ocean. Additionally, it is also an important aquacultural and ecological species [24,25]. The density of swimming crabs is usually high in most food-rich habitats [24]. It is vulnerable to intense fighting and cannibalism due to its aggressive nature [26,27], resulting in high mortality and fluctuations in the population [3]. Previous studies have found that cannibalism can attributed to lack of food, crowded space, and stresses generated by an unhealthy environment [28,29]. Territory may have potential in regulating cannibalism of territorial crustaceans since it has been indicated to affect competition [25]. However, the systematic underlying patterns of how these behaviors associated with aggression interact with territory are rarely examined [30]. Therefore, in order to better understand and predict the population fluctuations of *P. trituberculatus*, we quantitatively analyzed how the territorial behaviors of occupants are influenced by the different number of intruders in an indoor observation system. We predicted that with the increasing intruders, the occupant will shrink its territory size and reduce the activity associated with territory protection. The results of this study will improve our understanding of the territorial behavior of crustaceans and the predictive power regarding their population fluctuations.

## 2. Materials and Methods

### 2.1. Animal Collection and Maintenance

The experiment was carried out from August to September of 2020 at the Key Laboratory of Mariculture, Ministry of Education, Ocean University of China, Qingdao, China. A total of 130 male swimming crabs (carapace widths (CW) = 9.33 ± 1.42 cm; wet weights = 112.63 ± 26.67 g) were collected from an aquaculture facility in Jiaozhou, Shandong Province. Crabs were acclimatized in separate aquaria (40.50 L, 45 × 30 × 30 cm) for one week. The temperature of the filtered seawater (80 µm) was 22 ± 1 °C, and the salinity was 30. The photoperiod was 12:12 light/dark. Aquaria were aerated continuously, and 1/2 of the water was exchanged daily. Crabs were fed with adequate Manila clams (*Ruditapes philippinarum*) each day. Excrement and leftover clams were removed from the aquaria 4 h after feeding.

### 2.2. Experimental Design and Procedure

**Figure 1:**
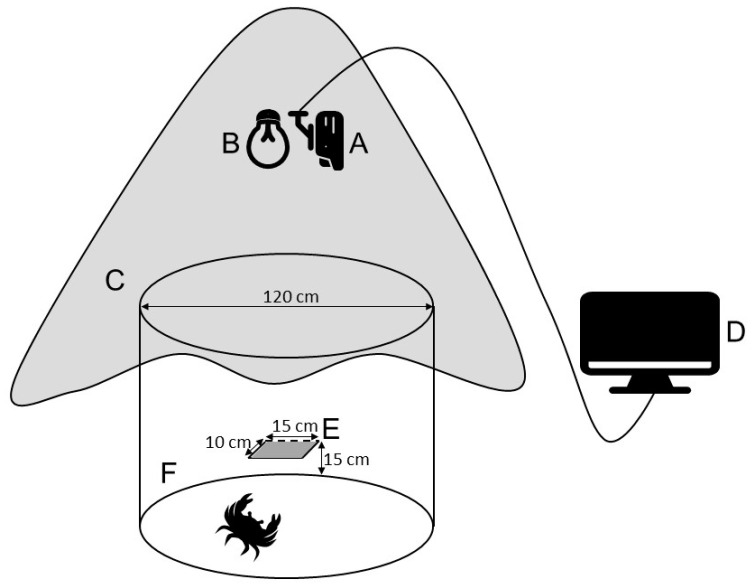
Diagram of an indoor behavioral observation system. (**A**) infrared camera, (**B**) light source, (**C**) shading cloth, (**D**) monitor, (**E**) shelter, and (**F**) experimental aquarium.

An indoor behavioral observation system was set up in a quiet room (Figure 1) [25]. The system consisted of an infrared camera (DS-2CD864, Hikvision, Hangzhou, China), a monitor (233i, Philips, Zhongshan, China), a cylindrical experimental aquarium (white PVC, diameter = 120 cm; height = 100 cm), light source (5W, Philips, Zhongshan, China), shading cloth (white polyester, 2.5 m × 2.5 m), and shelter (black PVC board, 15 cm × 10 cm). The camera and light source were installed 1.0 m above the experimental aquarium. The aquarium was filled with 340 L of filtered seawater (depth = 30 cm). The shelter was fixed horizontally on the wall of the aquarium 15 cm from the bottom. To ensure that the system was not affected by external interference, the whole system was covered with a shading cloth. During recording, the temperature and salinity were the same as during acclimatization, but the aquarium was not aerated.

After 1 week of acclimatization, crabs with intact appendages in the intermolt stage were selected. Three treatments were established, and the groups had 2, 4, and 8 crabs, denoted as group low (L), medium (M), and high (H), respectively. The crabs whose carapace widths differed by less than 5% were assigned to the same group to reduce the impact of size differences (group L: CW = 9.09 ± 1.17 cm; group M: CW = 8.87 ± 1.76 cm; group H: CW = 9.82 ± 1.90 cm). One crab was randomly selected from each group and designated as the occupant, and the remaining individuals were designated as intruders. The numbers of intruders in the three treatments were 1, 3, and 7, respectively. Crabs were not fed for 24 h before the experiment. A white number was painted on the carapace of each crab for identification (Liquitex HB, Liquitex Artist Materials, Athens, OH, USA). Each treatment had eight replicates, and each crab was only used once in the experiment.

At the beginning of the experiment, ten Manila clams were randomly added into the aquarium as prey to stimulate the crab to establish a territory [25]. An occupant crab was then added to the aquarium, allowing it to move freely. The intruders were placed gently into the aquarium after 24 h of introducing an occupant. After that, the experiment continued running for 1 h [20]. Each experiment lasted 25 h.

### 2.3. Data Acquisition and Quantification

Only the videos taken after the addition of the intruders (1 h) were analyzed to quantify the territorial behavior of occupants.

#### 2.3.1. Territory Size

To assess territory size, one picture was captured from the video every minute, resulting in 60 total frames being obtained from each video. The coordinates of occupants during that hour were determined using the GetData Graph Digitizer 2.26, and these data were used for the calculation of the territory size [25]. Using Matlab 2019, the size of each point was linked up, the territory sizes were calculated using the 95% fixed kernel method, and the least-squares cross-validation value was used for smoothing [31,32]. The bivariate density for each normal distribution at any given point was calculated (Formula 1) [31,32,33] as

(1) $$D_{n}=\frac{1}{2\pi \sigma_{x}\sigma_{y}\sqrt{1-\rho^{2}}}\times \exp\left\{-\frac{1}{2\left(1-\rho^{2}\right)}\left[{\left(\frac{x-\mu_{x}}{\sigma_{x}}\right)}^{2}+{\left(\frac{y-\mu_{y}}{\sigma_{y}}\right)}^{2}-2\rho \left(\frac{x-\mu_{x}}{\sigma_{x}}\right)\left(\frac{y-\mu_{y}}{\sigma_{y}}\right)\right]\right\}$$

In this formula, $\sigma_{x}$ and $\sigma_{y}$ is the stand deviation of the coordinates. $\mu_{x}\;$ and $\mu_{y}\;$ are the coordinates of the center of the experimental aquarium. *ρ* is the X-Y covariance.

#### 2.3.2. Defense Time and Occupying Time

Defense time, which is equal to the time spent on patrolling (move in its territory) and expelling intruders (dash to the intruder, direct its cheliped, and fight with it), was calculated [14,34]. Besides, time occupying shelter (more than 2/3 of the carapace under the shelter) of each occupant was determined [25].

#### 2.3.3. Quantification of Territorial Behaviors, Bouts, and Prior Residence Effect

The total number of territorial behaviors in occupants, which includes aggressive dash, aggressive wave, and fight, was calculated using the EthoVision XT 10 software, and each behavioral component was identified (Table 1) [34]. Furthermore, we counted the number of bouts initiated by the occupant and intruder in each replicate separately, as well as the winning percentage for each type of crab. One bout is defined as the process of crabs moving to, contacting, and retreating. The crab that caused its opponent to retreat repeatedly in a bout was considered the winner [29]. The crab gained a “dominance sign” when it caused its opponent to retreat in the last bout [20]. If the occupant accumulated six consecutive dominance signs and did not lose in any fights during the next 10 min, it was categorized as exhibiting the prior residence effect. If the occupant was defeated while accumulating dominance signs, the sign returned to zero. If the occupant did not gain 6 consecutive dominance signs in the selected period, it was categorized as not showing the prior residence effect. Besides, if it was difficult to tell winner and loser apart in several bouts, the occupant was categorized as “not clear” [20]. Finally, the number of bouts between the occupant and intruders at different distances from the shelter was calculated. For the convenience of analysis, the distances were measured in units of 25 cm.

#### 2.3.4. Dominance Hierarchy

Dominance hierarchy was analyzed using videos in high density group (1 occupant + 7 intruders, 1 h × 8 replicates). The number of winning bouts of each individual was counted, and a matrix of wining bouts was established. *N_ij_* is the number of bouts in which individual *i* defeated individual *j*. *M_ij_* is the number of bouts in which individual *i* was defeated by individual *j*. *P_ij_* is the winning percentage of individual *i* when it fought against individual *j*. David’s Score of individual *i* in this group was calculated (Formula (2)) using the following

(2) $$DS=w+w_{2}-l-l_{2}$$

(3) $$w=\sum P_{ij}$$

(4) $$w_{2}=\sum (P_{ij}\times N_{ij})$$

(5) $$l=\sum \left(1-P_{ij}\right)$$

(6) $$l_{2}=\sum (1-P_{ij})\times \sum M_{ij}$$

where *w* (3) is the sum of the winning percentages of individual *i* in the fights with each individual; *w_2_* (4) is the weighted sum of the individual’s *w*; *l* (5) is the sum of failure rate of individual *i* in the fights with each individual; and *l*_2_ (6) is the weighted sum of the individual’s *l* value [16].

#### 2.3.5. Territorial Behavior Score

The occupant’s territory size, total number of territorial behaviors, defense time, number of bouts, and winning percentage were input into the principal component model. The first component (PC1) was designated as the “territorial behavior score” because it reflected the territoriality of occupants [25,35].

### 2.4. Statistical Analysis

All data were analyzed using SPSS Statistics 24.0. A chi-square test was used to analyze the proportion of prior residence effect. A Spearman test was used to identify correlations in the number of bouts between occupant versus intruder and distance from the shelter. The generalized linear mixed model (GLMM) was used to analyze the territory size, defense time, occupying time, number of territorial behaviors, the total number of bouts in the selected period and bouts initiated by occupant, the winning percentage of occupant, the difference of David’s Score between occupants and intruders, and the territorial behavior score of occupant. When analyzed in GLMM, a binomial distribution was used for the number of bouts initiated by the occupant and the winning percentage of occupant; a skewed distribution was used for territory size, defense time, occupying time, David’s Score between occupants and intruders, and territorial behavior score; and a negative binomial distribution was used for number of territorial behaviors and bouts. For models with skewed distributions, the residuals were checked to for normal distribution to assess model fit. Models with binomial error structures were checked for overdispersion. For number of territorial behaviors and bouts, negative binomial distributions were chosen to deal with overdispersion. When analyzing the difference of David’s Score between occupants and intruders, the ID of crabs and number of parallel groups were included as random factors, and the classifications of occupying crabs and intruders were taken as fixed factors. In the rest of the analyses, the ID of crabs was included as a random factor, and the number of intruders was taken as a fixed factor. Post hoc comparisons between different treatments were conducted with the calculation module in SPSS. For all tests, *p* < 0.05 was considered to be statistically significant.

## 3. Results

### 3.1. Territory Size

**Figure 2:**
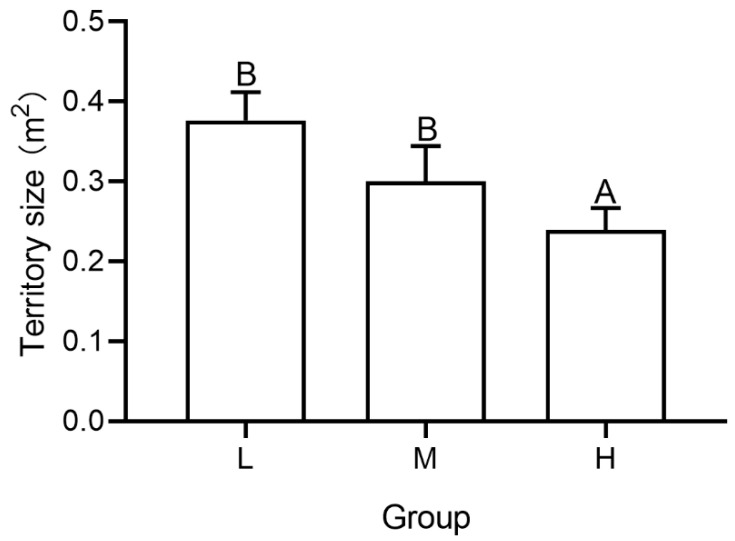
Territory size of occupant in groups with different number of intruders. Capital letters represent significant differences between each group (*p* < 0.05).

The territory size of occupants was significantly affected by the number of intruders (*F* = 3.851, *p* = 0.034, Figure 2). The territory size of occupants in group H was significantly smaller than those of group L (*t* = 4.173, *p* < 0.001) and group M (*t* = 6.146, *p* = 0.047).

### 3.2. Defense Time and Occupying Time

**Figure 3:**
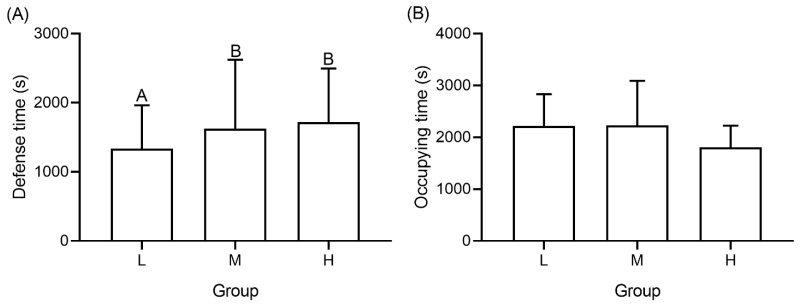
Defense time (**A**) and occupying time (**B**) of occupant in groups with different number of intruders. Capital letters represent significant differences between each group (*p* < 0.05).

With an increased number of intruders, the defense time of occupants increased significantly (*F* = 5.217, *p* = 0.044, Figure 3A), but the occupying time did not (*F* = 2.364, *p* = 0.729, Figure 3B). The defense time of occupants in group M (*t* = 3.129, *p* = 0.011) and group H (*t* = 4.467, *p* = 0.016) was significantly longer than in group L.

### 3.3. Quantification of Territorial Behaviors, Bouts, and Prior Residence Effect

**Figure 4:**
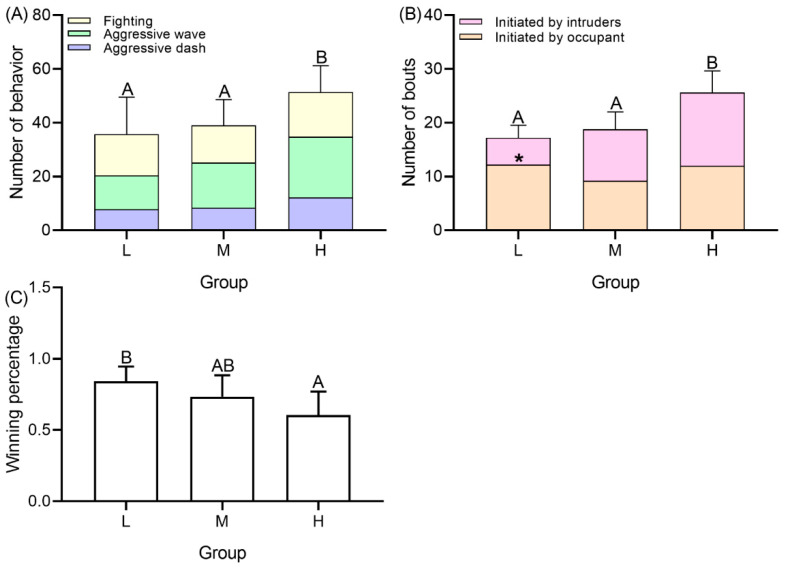
Number of territorial behaviors (**A**), number of bouts (**B**) and winning percentage (**C**) of occupant in groups with different number of intruders. Capital letters represent significant differences between each group (*p* < 0.05). Asterisks (*) represents significant differences between bouts initiated by intruders and occupant (*p* < 0.05).

The number of intruders significantly affected the number of territorial behaviors (*F* = 4.868, *p* = 0.039, Figure 4A), bouts (*F* = 8.322, *p* < 0.001, Figure 4B), and the winning percentages of occupants (*F* = 5.297, *p* = 0.047, Figure 4C). With an increased number of intruders, the number of territorial behaviors increased significantly, and the number in group H was significantly more than group L (*t* = 4.739, *p* = 0.002) and group M (*t* = 5.551, *p* = 0.029). The number of bouts between occupants and intruders also increased significantly, and the number was significantly higher in group H than group L (*t* = 6.869, *p* = 0.003) and group M (*t* = 7.748, *p* = 0.044). In group L, the number of fights initiated by occupants was significantly higher than those initiated by intruders (*F* = 8.517, *p* = 0.024).

Among the three treatments, the proportion of the occupants in group L and group M exhibiting the prior residence effect was higher (Table 2). The prior residence effect in group H was not obvious (Table 2).

### 3.4. Dominance Hierarchy

**Figure 5:**
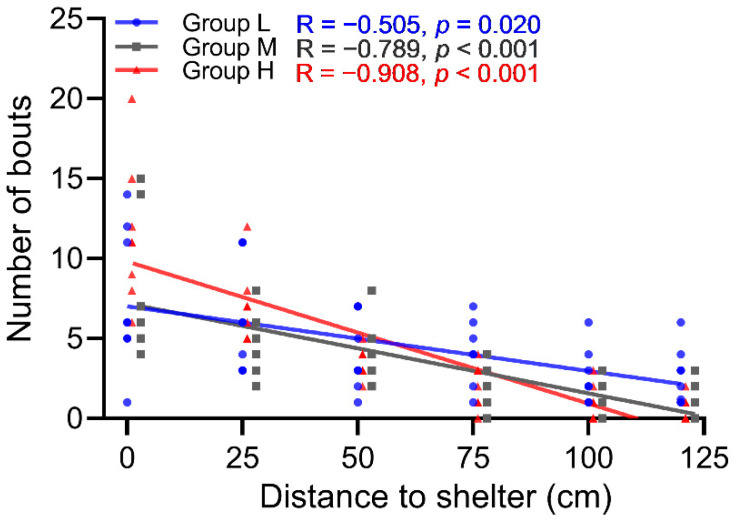
Spearman correlations between distance to shelter and number of bouts.

**Figure 6:**
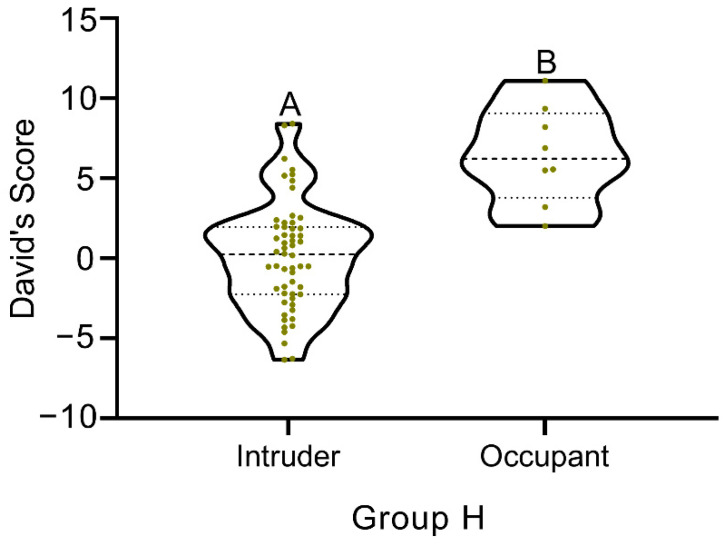
Comparison of David’s Score of occupant and intruder in group H. Capital letters represent significant difference between intruder and occupant (*p* < 0.05).

In all three treatments, there were significantly negative correlations between the number of bouts between the occupant versus intruder and distance from the shelter (Figure 5). With increased distance from the shelter, the number of bouts in all groups significantly decreased, but the decreases in group M and group H were much more pronounced compared to group L (group M: R = −0.789, *p* < 0.001, *n* = 8; group H: R = −0.908, *p* < 0.001, *n* = 8; group L: R = −0.505, *p* = 0.02, *n* = 8). Among the eight repeats in group L, five occupants attained the highest David’s Score, two occupants ranked second, and one occupant ranked third (Figure 6). In group L, the David’s Scores of occupants were significantly higher than those of intruders (*t* = 24.236, *p* < 0.001, *n* = 8, Figure 6).

### 3.5. Territorial Behavior Score

**Figure 7:**
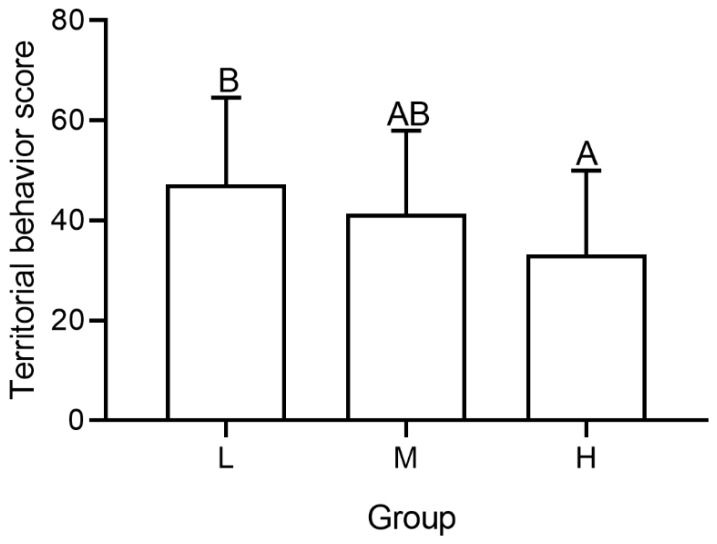
Territorial behavior score of occupant in groups with different number of intruders. Capital letters represent significant difference between each group (*p* < 0.05).

The territorial behavior score (i.e., PC1) included dimensionality of the territory size, total number of territorial behaviors (aggressive dash, aggressive wave, and fighting), defense time, number of bouts, and winning percentage of occupants, and explained 54.8% of the total variance (Table 3). The territorial behavior scores of occupants were significantly affected by intruder number (*F* = 19.462, *p* = 0.038, Figure 7). With an increasing number of intruders, the territorial behavior score of occupants decreased, and the score of group H was significantly lower than group L (*t* = 5.432, *p* < 0.001).

## 4. Discussion

When the number of intruders increased, the number of territorial behaviors and bouts increased (Figure 4A). Meanwhile, the proportion of fights initiated by occupants decreased as the number of intruders increased (Figure 4B). These results demonstrate the increasing aggression by the occupant and costs of expelling. With increased intruders, the territory size of occupants decreased (Figure 2), which likely reflects the costs to benefits balance of occupying a territory, that is, in highly competitive scenarios it is no longer beneficial to occupy a large territory [36]. Moreover, the proportion of individuals that exhibited the prior residence effect decreased as the intruder number increased (Table 2), suggesting that the costs of occupying correspondingly enhanced. In high density group that has 7 intruders, the occupant may not have the expected advantage in competition with intruders due to the high costs. These results were aforementioned with the territorial behavior score-when the number of intruders increased, the scores significantly decreased (Figure 7), indicating the territoriality of occupants became weaker. The results are in agreement with the fact that *P. trituberculatus* is an energy maximizing species–meaning it acquires energy and reduces costs of energy acquisition whenever possible [8]. Defense time increased when the number of intruders increased (Figure 2 and Figure 3A), which is also consistent with the response to the change of external conditions of energy maximizing [14]. However, in our study, the winning percentage of occupants did not significantly decrease with the increase in the number of intruders (Figure 4C), consistent with studies on behaviors (*Allopetrolisthes spinifrons*) [37]. We concluded that the reduced territory size may not be a forced choice caused by pressure from intruders, but a voluntary abandonment of part of the territory by the occupant.

Shelter is the core of the territory and the main target of competition for resources in the wild [38]. For example, when an intruder cannot be evicted from a territory, fiddler crabs (*Leptuca leptodactyla*) will preferentially protect their cave shelter instead of territory [39]. In our study, when the number of intruders increased, the number of bouts farther away from the shelter decreased (Figure 5), which reflected a prioritization of the shelter. Despite this, the occupying time of the occupant did not increase as the intruder count increased (Figure 3B), which can be explained by the fighting that frequently occurred in the vicinity of the shelter causing the occupant to lose and regain shelter intermittently. Similar to reports studying king crab (*Paralithodes Camtschaticus*) and spider crab (*Maja squinado*) [40,41], adjusting the population density and total number of shelters may be an effective way to reduce the territorial disputes among *P. trituberculatus*, in other words, to diminish their territoriality.

In a population, the dominance hierarchy is often associated with preferential access to resources [6,42,43]. In studies of territorial behavior, occupants usually have higher levels on dominance hierarchies than intruders [16]. The top five David’s Scores were gained by occupants in high density group, and those occupants also exhibited the prior residence effect. That is to say, those five occupants had absolute dominance of their territory (Figure 6, Table 2), which is similar to that found in territorial competition in gobies (*Elacatinus prochilos*) and crayfish (*Austropotamobius pallipe*s), respectively [18,42]. Dominators occupy larger and better territories, and expel subordinates from it, which negatively affects the growth of the subordinate and even the survival rate in the population. All the dominator–subordinate dualities can also be combined to form the dominance hierarchy of the population [44]. In research of population of marine biology, it is significant to understand the influence of the dominance hierarchy on territorial behavior. For example, it was found that the density and yield predictions of Norwegian lobsters (*Nephrops norvegicus*) could be assessed using the dominance hierarchy [45]. In addition, an individual’s rank on the dominance hierarchy is usually related closely to its size [46]. Classification in crustacean cultures can therefore be based on individual sizes, this can be utilized with the principal goal to reduce differences in the dominance hierarchy among individuals, which is also an effective means to improve the survival rate and yield by adjusting territorial behavior.

Territorial behavior of intruders and occupants also plays an important role in regulating population density [15,47,48]. In the wild, a region with abundant resources and few competitors will encourage animals to aggregate. When the conspecific density becomes high, territories the individual owns will shrink and overlap, leading to an intensified territorial dispute [49], which causes frequent emigration, fight and cannibalism and reduces density and competitive pressure until the density stabilizes [47]. In our study, the swimming crabs were unable to emigrate to reduce the density due to limited observation areas, therefore, territorial competition was the only means for intruders to acquire territory and for the occupant to maintain their territory. This also explained why territory size decreased and the number of territorial behaviors and bouts increased when the number of intruders was increased (Figure 4A, 4B). The high density promotes animals to invade nearby territories and engage in fierce fighting, accompanied by increased mortality and reduced growth rates [50,51]. In extreme cases, fish give up their territory when the density is too high, and they cannot emigrate [52]. However, relevant results have not been reported in crustaceans, and the trends of decreasing numbers of territorial behaviors were not observed in the multiple intruder treatments in our study (Figure 3A). A possible reason is that swimming crabs live mainly on the seabed, a two-dimensional surface, which enhances the importance of territory and the intensity of territorial dispute, and male crabs reluctant to abandon their territory [53]. From the perspective of territorial behavior, a high-density crab population may be unstable and therefore unlikely to last for long in the community.

## 5. Conclusions

In our study, when the number of intruders increased, the number of a swimming crab’s territorial behaviors increased, but their territory size decreased. When the density of crabs was high, there was a clear dominance hierarchy. According to the results, due to the intensification of territorial competition, an increase in density may lead to a decrease in survival rate. However, the territorial behavior of crabs is greatly influenced by vision [54], thus increasing the complexity in the seabed, such as establishing artificial shelters or planting algae, which may be an effective means by which to limit territorial behavior [46,55]. Therefore, optimizing the habitats in marine ranching using environmental enrichment will ensure the densities of crabs rationally increases by reducing conflict over territory [56]. The specific approaches to optimizing environments need to be examined further to assist in the exploration of population dynamics of swimming crabs.

## Figures and Tables

**Table 1:** Description of territorial behavior.

Territorial Behavior Type	Description
Aggressive dash	One crab makes a dash at the other without cheliped-waving.
Aggressive wave	One crab directs its cheliped, waving to the other.
Fighting	Crabs approach each other with their chelipeds motionless. Then, their chelipeds make manus–manus contact and a little pushing follows.

**Table 2:** Results of chi-square tests that examined the quantity and proportion of occupants showing prior residence effect under different number of intruders.

Group	Whether Prior Residence Effects Exist or Not			Proportion of Prior Residence Effect	χ^2^	*p*
	Yes	No	Not Clear			
L	7	0	1	0.875	4.500	**0.034**
M	6	1	1	0.750	6.250	**0.044**
H	5	1	2	0.625	3.250	0.197

Note: *p*-Values in bold indicate significant results (*p* < 0.05).

**Table 3:** Component loadings for the first principal components factor. The first principal components factor of each crab represents its territorial behavior score.

Component Loadings	PC1
Territory size	0.602
Total number of territorial behaviors	0.417
Defense time	0.348
Number of bouts	0.674
Winning percentage	0.359

## Data Availability

The data presented in this study are available in the article. Further information is available upon request from the corresponding author.
